# Increasing the efficiency of CRISPR/Cas9-mediated genome editing in the citrus postharvest pathogen *Penicillium digitatum*

**DOI:** 10.1186/s40694-024-00179-0

**Published:** 2024-07-13

**Authors:** Carolina Ropero-Pérez, Jose F. Marcos, Paloma Manzanares, Sandra Garrigues

**Affiliations:** Food Biotechnology Department, Instituto de Agroquímica y Tecnología de Alimentos (IATA), Consejo Superior de Investigaciones Científicas (CSIC), Catedrático Agustín Escardino Benlloch 7, Paterna, Valencia, 46980 Spain

**Keywords:** Filamentous fungi, Gene targeting, Episomal expression vector, Non-model fungi

## Abstract

**Background:**

*Penicillium digitatum* is a fungal plant pathogen that causes the green mold disease in harvested citrus fruits. Due to its economical relevance, many efforts have focused on the development of genetic engineering tools for this fungus. Adaptation of the CRISPR/Cas9 technology was previously accomplished with self-replicative AMA1-based plasmids for marker-free gene editing, but the resulting efficiency (10%) limited its practical implementation. In this study, we aimed to enhance the efficiency of the CRISPR/Cas9-mediated gene editing in *P. digitatum* to facilitate its practical use.

**Results:**

Increasing the culture time by performing additional culture streaks under selection conditions in a medium that promotes slower growth rates significantly improved the gene editing efficiency in *P. digitatum* up to 54–83%. To prove this, we disrupted five candidate genes that were chosen based on our previous high-throughput gene expression studies aimed at elucidating the transcriptomic response of *P. digitatum* to the antifungal protein PdAfpB. Two of these genes lead to visual phenotypic changes (PDIG_53730/*pksP*, and PDIG_54100/*arp2*) and allowed to start the protocol optimization. The other three candidates (PDIG_56860, PDIG_33760/*rodA* and PDIG_68680/*dfg5*) had no visually associated phenotype and were targeted to confirm the high efficiency of the protocol.

**Conclusion:**

Genome editing efficiency of *P. digitatum* was significantly increased from 10% to up to 83% through the modification of the selection methodology, which demonstrates the feasibility of the CRISPR/Cas9 system for gene disruption in this phytopathogenic fungus. Moreover, the approach described in this study might help increase CRISPR/Cas9 gene editing efficiencies in other economically relevant fungal species for which editing efficiency via CRISPR/Cas9 is still low.

**Supplementary Information:**

The online version contains supplementary material available at 10.1186/s40694-024-00179-0.

## Background

Filamentous fungi are a major threat to human and animal health, crop production and food safety [1]. However, fungi can also serve as eukaryotic models in both fundamental and applied research and as biofactories for the biotechnological production of a broad source of metabolites, enzymes and other proteins of commercial applications [2]. In this context, genetic and metabolic engineering are effective technologies for elucidating gene function and also increasing production levels while minimizing unwanted by-products.

*Penicillium digitatum* is a fungal plant pathogen that causes the green mold disease in harvested citrus fruits, producing very important economic losses worldwide [3]. Additionally, *P. digitatum* has been demonstrated as an efficient fungal cell factory for the homologous and heterologous production of the so-called fungal antifungal proteins (AFPs) [4, 5] and other cysteine-rich proteins [6]. Therefore, due to the economic and social relevance of this fungus, many efforts have focused on the characterization of the genetic and molecular mechanisms involved in its pathogenicity and virulence, resistance to antifungal compounds and protein production [4, 6–16].

One of the limiting factors for effectively disrupting genes in filamentous fungi, including *P. digitatum*, has traditionally been low frequencies of homologous recombination (HR) at a target locus [17]. A breakthrough that improved homology recombination rates in (some) fungi evolved through the disruption of genes involved in the non-homologous end joining (NHEJ) DNA repair pathway by *ku70* and/or *ku80* gene knock-down. However, this approach is far from being optimal, since in some cases the inactivation of NHEJ has been associated with increased vulnerability to DNA damaging and decreased strain fitness [18]. In *P. digitatum*,* ku70* deletion facilitates homologous recombination but at the expense of increasing the temperature sensitivity of the fungus, which results in a detrimental effect in axenic growth and conidia production [4]. Thus, the utilization of *P. digitatum* NHEJ-deficient strains does not seem to be the most appropriate approach to further develop this fungus for biotechnological purposes.

Lately, the CRISPR/Cas9 technology has emerged as a cutting-edge genome editing tool to overcome the low homologous integration frequencies of filamentous fungi [19]. The Cas9 endonuclease, driven to target genes by a single guide RNA (sgRNA) that forms a sequence-specific RNA complex, produces double-strand breaks (DSBs) that induce the NHEJ response for their repair. In this process, random mutations can be introduced, leading to frameshift variants that produce non-sense sequences or premature stops codons, hence blocking the proper translation of the mRNA [20]. Recently, our group has successfully implemented this technology in *P. digitatum* with a reported genome editing efficiency of 10% by using non-integrative AMA1-based self-replicating plasmids that contain the required CRISPR/Cas9 machinery [21]. After DNA editing, the subsequent curing of the plasmid renders a marker-free strain that can be subjected to successive rounds of mutation. However, the editing rate remains low for practical purposes, and an optimization of the already described protocol for the efficient genetic modification of *P. digitatum* through CRISPR technology is urgently needed. In this study, we show increased genome editing efficiency of *P. digitatum* wild-type strain from the previously reported efficiency rate of 10% to up to 83% by means of modifying the transformant selection methodology. The optimized protocol for the efficient CRISPR/Cas9-mediated genome editing in *P. digitatum* is presented and discussed.

## Results and discussion

The application of the CRISPR/Cas9 technology has been demonstrated in filamentous fungi, [22–24] with very different degrees of genome editing efficiencies, ranging from 1% in *Aspergillus carbonarius* [25] up to almost 100% efficiency in *Aspergillus niger* [26, 27]. In the phytopathogenic fungus *P. digitatum*, the CRISPR/Cas9 system has been demonstrated for the first time by our group, but with genome editing efficiencies of just 10% [21], which we consider an important limiting factor for the efficient genetic modification of the fungus. Therefore, protocol optimization for CRISPR/Cas9 gene editing in *P. digitatum* is required.

The modification of genes that lead to visual phenotypic changes such as color change or (in)ability to grow on several substrates easily leads to the identification of the colonies that underwent the genetic modification by visual inspection directly on the transformation and/or selection plates. This was the strategy of choice while implementing the CRISPR/Cas9 technology in *P. digitatum* for the first time [21]. In this previous work, we targeted the *wetA* gene, which encodes a conidiophore development-related transcription factor whose disruption generated white, cotton-like mutant colonies [21, 28]. However, genetic modification of the majority of genes present in a fungal organism does not lead to easily distinguishable phenotypes, which impedes the identification of the mutant strains by phenotyping. Therefore, we aimed to increase the gene editing efficiency of the CRISPR/Cas9 system in *P. digitatum* in a two-stage approach. First, by disrupting genes associated with phenotypes that can be easily observed to improve the gene-editing protocol in a visual and fast-screening manner. And second, by demonstrating the utility of the optimized protocol with other genes that are not initially associated with visually discernible phenotypes.

For this purpose, we chose five gene candidates that were differentially expressed based on our high-throughput gene expression studies aimed at elucidating the killing mechanism of the AFP PdAfpB against the citrus postharvest pathogen *P. digitatum* [16], which are summarized in Table 1. AFPs are small, cationic, cysteine-rich proteins secreted by ascomycete fungi that stand out as promising antifungal biomolecules to be applied in agriculture, food and clinic to fight fungal pathogens [29, 30].

**Table 1:** Examples of differentially expressed genes in *P. digitatum* after PdAfpB treatment [16]. Gene candidates chosen for CRISPR/Cas9 efficiency optimization are highlighted in bold

Gene ID	Gene annotation	(Predicted) function
**PDIG_53730**	***pksP/alb1***	**Conidial pigment polyketide synthase**
PDIG_54070	*abr1/brown 1*	Conidial pigment biosynthesis oxidase
PDIG_54080	Hypothetical protein	Multicopper oxidase
PDIG_54090	*arp1*	Conidial pigment biosynthesis scytalone dehydratase
**PDIG_54100**	***arp2***	**Conidial pigment biosynthesis reductase**
PDIG_54110	*ayg1*	Conidial pigment biosynthesis protein
**PDIG_68680**	***dfg5***	**Cell wall glycosyl hydrolase**
**PDIG_56860**	**Glycoside hydrolase**,** family 47**	**Mannosyl-oligosaccharide 1**,**2-α-mannosidase**
**PDIG_33760**	**Hydrophobin**	**Hydrophobin**

### Optimization of CRISPR/Cas9 genome editing in *P. digitatum* through the disruption of genes rendering altered conidia pigmentation

**Fig. 1:**
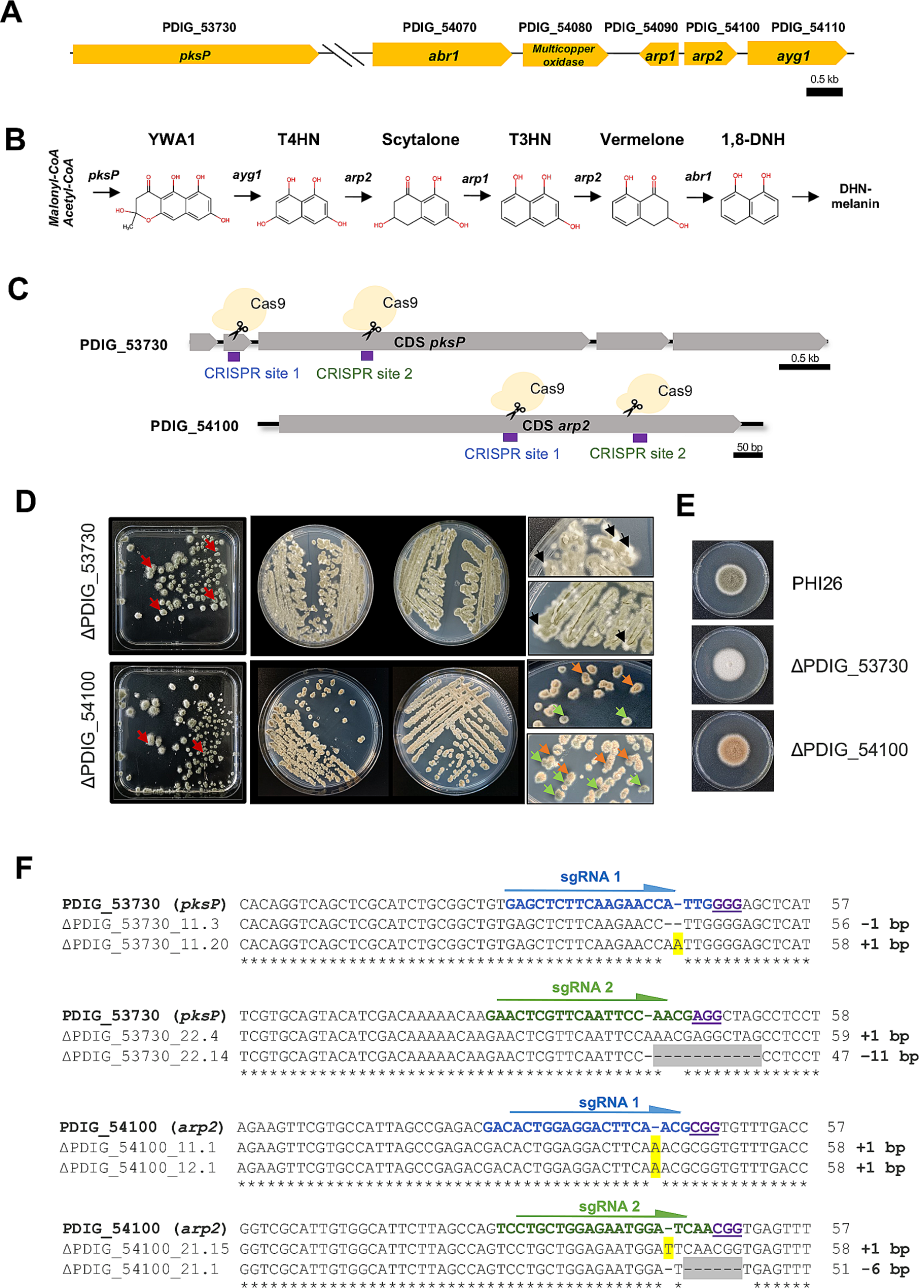
Application of CRISPR/Cas9 to target *pksP* (PDIG_53730) and *arp2* (PDIG_54100) genes of the DHN-melanin biosynthetic pathway. (**A**) Genomic organization of the DHN-melanin biosynthesis gene cluster in *P. digitatum*. (**B**) DHN-melanin pathway predicted for *P. digitatum.* (**C**) Schematic representation of sgRNA target sites designed for the disruption of *pksP* and *arp2* genes by CRISPR/Cas9. (**D**) Transformation results of *P. digitatum* after growth in the initial PdMM selection plate (left) and subsequent striking of transformed colonies in selective medium (right). Red arrows represent examples of green colonies that were randomly chosen from the primary transformation plates. Black and brown arrows point to albino and brownish colonies appearing after colony streaking, respectively. Green arrows point to non-edited colonies. (**E**) Colony morphology of parental wild-type *P. digitatum* (PHI26 strain) and two individual disruption mutants (Δ*pksP* and Δ*arp2*) after 5 days of growth on solid PDA plates. (**F**) Nucleotide sequence alignments of Sanger sequencing results for *pksP* and *arp2* mutants compared to the parental sequences. Multiple sequence alignments were effectuated with the Geneious Aligner (Geneious Prime^®^ 2023.0.4). Protospacer adjacent motif (PAM) sequences are underlined and 20-bp protospacers are indicated in blue (sgRNA 1) and green (sgRNA 2)

Among the genes significantly repressed upon PdAfpB treatment were those belonging to the 1,8-dihydroxynaphtsalene (DHN)-melanin biosynthesis cluster (Table 1). This cluster was previously characterized in *P. digitatum* [31], describing five genes involved in DHN melanin biosynthesis: *pksP* (PDIG_53730), *abr1* (PDIG_54070), *arp1* (PDIG_54090), *arp2* (PDIG_54100) and *ayg1* (PDIG_54110) (Fig. 1A). Mutants of these genes render colonies with various degrees of altered conidial pigmentation. We targeted two of them, *pksP* and *arp2*, to allow fast screening of potentially successful CRISPR mutants. The *pksP* is a polyketide synthase that catalyzes the conversion of acetyl-CoA and malonyl-CoA to heptaketide napthopyrone (YWA1) (Fig. 1B). In contrast, *arp2* encodes a reductase putatively involved in two different steps of the melanin biosynthetic pathway: conversion of 1,3,6,8-tetrahydroxynaphthalene (T4HN) to scytalone and reduction of 1,3,8-trihydroxy-naphthalene (T3HN) intermediate to vermelone (Fig. 1B). Disruption of *pksP* and *arp2* in *P. digitatum* causes color change from the original green colonies to white (*albino*) and reddish-brown colonies, respectively [31]. Two different sgRNAs where designed to target each of these two genes (Supp. Table S1) at different locus positions. Their location within the gene sequences is shown in Fig. 1C. After 7 days post-transformation with the episomal self-replicating pLM-AMA15.0 plasmid derivatives, we did not detect phleomycin resistant colonies that had incorporated the plasmid with the expected color-changing phenotype in the protoplast transformation plates (square plates) (Fig. 1D). Therefore, between 16 and 27 randomly selected green colonies (Table 2) were transferred to phleomycin-containing *P. digitatum* Minimal Medium (PdMM) plates (see Fig. 1D, red arrows as an example) and were allowed to grow for additional 3–5 days at 25 ºC (Fig. 1D, round Petri dishes). In the previously reported protocol, randomly chosen transformants were picked from the transformation plates to selective potato dextrose agar (PDA) plates in order to discard false positive colonies, and after 2 days of incubation, these were directly transferred to non-selective PDA plates to accelerate plasmid curation [21]. However, the rapid fungal growth achieved in complete PDA medium together with the short incubation time applied (2 days) resulted in no color-changing colonies in this selection step. In contrast, with longer incubation times (up to 5 days) in selective PdMM, in which the fungal growth rate for *P. digitatum* is substantially reduced, a large number of gene edited colonies appeared, which were easily distinguishable from the parental-related green phenotype (Fig. 1D, zoomed images), highlighting the importance of selecting optimal growth media and incubation times for Cas9-induced activity. In the case of *pksP* gene disruption with sgRNA1, 100% of the strikes performed from the originally 16 transformed colonies with green phenotype resulted in a mixture of easily distinguishable green/white phenotypes in the second round of selection (Table 2). Similarly, in the case of *pksP* gene disruption with sgRNA2, 70% of the 20 green colonies randomly chosen resulted in mixed phenotypes. In the case of *arp2* gene disruption with both sgRNA1 and sgRNA2, 100% of the randomly chosen colonies resulted in mixed green/brownish phenotypes after the second round of selection (Table 2). After this selection round in PdMM, colonies showing the expected phenotypes of altered pigmentation (Fig. 1E) where transferred to selective 24-well PDA plates and their genomic DNA was isolated and sequenced for molecular confirmation (Fig. 1F). Sequencing results demonstrated that between 91 and 100% of the sequenced strains had nucleotide insertions or deletions within the target gene sequences for both sgRNAs (Fig. 1F and Supp. Fig S1), resulting in gene frameshifts in most cases (Table 2).

**Table 2:** CRISPR/Cas9-mediated genome editing of target genes PDIG_53730 and PDIG_54100.

Target gene	sgRNA	Differential phenotype color after one round of selection (%)	Editing efficiency (%)	Type of mutation	Mutation frequency (%)	Indel size frequency (%)
PDIG_53730 (*pksP*)	1	16/16 (100%)	12/12 (100%)	Insertion	9/12 (75%)	+ 1 bp (75%)
				Deletion	3/12 (25%)	-1 bp (16.6%) -2 bp (8.33%)
	2	14/20 (70%)	10/11 (91%)	Insertion	3/10 (30%)	+ 1 bp (30%)
				Deletion	7/10 (70%)	-1 bp (50%) -3 bp (10%) -11 bp (10%)
PDIG_54100 (*arp2*)	1	17/17 (100%)	4/4 (100%)	Insertion	4/4 (100%)	+ 1 bp (100%)
				Deletion	0/4 (0%)	-
	2	27/27 (100%)	4/4 (100%)	Insertion	3/4 (75%)	+ 1 bp (75%)
				Deletion	1/4 (25%)	-6 bp (25%)

It is noteworthy that the type of mutation incorporated after NHEJ seems to be sgRNA-dependent. For example, in case of *pskP* gene, sgRNA1 led to 75% insertions, while sgRNA2 led mostly to gene deletions (70%). In case of *arp2*, the majority of mutations produced by sgRNA1 and sgRNA2 were insertions, with 100% and 75% frequency, respectively (Table 2).

Targeted mutagenesis via Cas9 is efficient at temperatures of 37 ºC and higher, yet it becomes gradually impaired at lower temperatures [32]. Therefore, the endonuclease activity of Cas9 is dependent on the temperature in which the target organism is growing. The optimal growth temperature of *P. digitatum* is 25 ºC, with increasing impaired growth at temperatures above 28 ºC [4, 33]. Consequently, the low gene editing efficiency (10%) previously obtained for this organism [21] could be attributed to a low nuclease activity of the Ca9 protein at the optimal temperature of this fungus. However, due to the sensitivity of *P. digitatum* to temperatures higher than 28 ºC, increasing the incubation temperature was not an option to improve the genome editing efficiency driven by Cas9. Instead, we decided to increase the time the CRISPR/Cas9 machinery containing self-replicative plasmids were inside the colonies to theoretically (i) allow for greater plasmid copy number within the cells, if possible, and (ii) give more time for the Cas9 endonuclease to generate the DSBs at this sub-optimal temperature of 25 ºC by changing from PDA-selective plates to PdMM-selective plates in the subsequent selection steps and by increasing incubation time. It is important to note, however, that the fungal metabolic activity in PdMM might be slower than in PDA, and therefore, plasmid copy number achieved in transformants grown on PdMM plates does not necessarily have to increase despite longer incubation times. Additionally, the efficiency of both Cas9 translation and sgRNA transcription may be influenced by the growth media, with selection pressure likely having an important role in CRISPR/Cas9 efficiency, as it would avoid plasmid curation and, therefore, promote CRISPR/Cas9 activity. In *Alternaria alternata*, prolongation of the culture time of the transformants on the primary transformation plates allowed the development of mutations over time [34], which already pointed to time as a key factor for gene editing by CRISPR/Cas9. However, in the case of *P. digitatum*, extending culture time for up to 10 days in the transformation plates only resulted in colony overgrowth without appearance of mutated non-melanized conidia (data not shown). In some *Aspergilli*, however, re-inoculation of transformants on selection plates after transformation increased the number of colored sectoring colonies, pointing to an increase in the CRISPR/Cas9 efficiency that, in any case, was not quantified [35]. In the case of the *pksP* and *arp2* genes in *P. digitatum*, just one additional streaking in selective PdMM increased the number of mutant colonies, which could be directly identified on the plates. Additionally, these strains underwent plasmid curation after four streaks on non-selective plates as previously described [21], losing their ability to grow in the presence of phleomycin, and with no phleomycin resistance cassette being detectable in their genomic DNA by PCR (Supp. Fig. S2).

### Validation of the optimized CRISPR/Cas9 protocol by disrupting genes that are not associated with visually discernible phenotypes

In the above-mentioned experiments, despite the increase in the number of *pksP* and *arp2* edited strains obtained, the amount of still non-edited colonies (green colonies) in the streaks was significantly higher than that of the edited ones (Fig. 1D). In order to further increase the possibilities to choose correct gene-edited strains in the case of no visually recognizable phenotypes, further selection steps on selective PdMM plates were applied under the hypothesis that additional culture rounds would further increase the editing efficiency driven by Cas9.

Apart from the above mentioned DHN melanin biosynthesis-related genes, three other genes were selected from those significantly repressed upon PdAfpB treatments in *P. digitatum*. PDIG_68680 is an orthologue of the *Saccharomyces cerevisiae DFG5* gene, which encodes a glycosylphosphatidylinositol (GPI)-anchored membrane protein required for cell wall biogenesis [36]. PDIG_56860 encodes an uncharacterized putative mannosyl-oligosaccharide 1,2-α-mannosidase in *P. digitatum.* Finally, PDIG_33760 encodes a hydrophobin protein with 74.5% sequence identity to the *Aspergillus fumigatus* spore hydrophobin RodA and a 57.1% identity to the characterized hydrophobin A of the phytopathogenic fungus *Penicillium expansum*, whose single disruption rendered a loss in fungal hydrophobicity and reduced conidia dispersion capability [37]. Initially, disruption of none of these genes has any recognizable phenotype change, contrary to *pksP* and *arp2*.

**Fig. 2:**
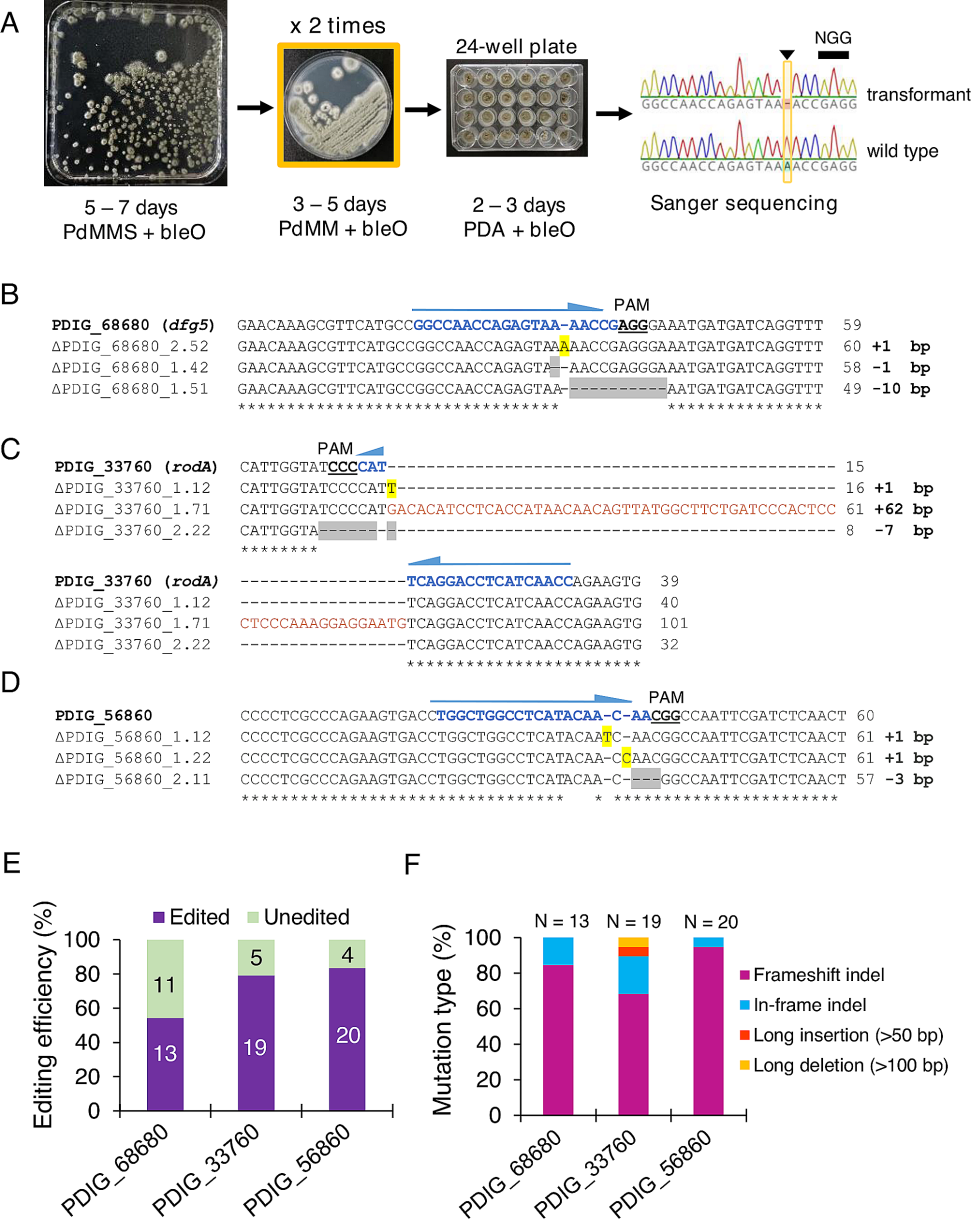
Application of CRISPR/Cas9 to target genes with no visual phenotype changes associated to their disruption (PDIG_68680, PDIG_33760 and PDIG_56860). (**A**) Representative images of the optimized transformation protocol steps followed for CRISPR/Cas9 editing and sequence confirmation. (**B**, **C, D**) Multiple sequence alignment of representative ΔPDIG_68680, ΔPDIG_33760 and ΔPDIG_56860 mutants aligned against the parental non-edited sequences. Alignments were effectuated with the Geneious Aligner tool (Geneious Prime^®^ 2023.0.4). Indels are highlighted in yellow (insertion) and grey (deletion) and a long insertion (> 50 bp) is indicated in orange. PAM sequences are underlined and the 20-bp protospacers are marked in blue. (**E**) Bar plot representing the gene editing efficiency (%) for each gene. The number of edited and un-edited transformants are indicated in each column (*n* = 24). (**F**) Bar plot showing the type of mutations identified for each targeted gene. The total number of transformants evaluated is indicated in each bar

In this case, one sgRNA was designed to target each individual gene, and two (instead of one) additional steps of transformant selection in PdMM plates were performed (Fig. 2A). Twelve isolated colonies coming from the primary transformation plates were randomly chosen and streaked into phleomycin-containing plates up to two times. After two rounds of selection, two isolated colonies from each streak were transferred to phleomycin-containing 24-well PDA plates. Genomic DNA of a total of 24 monosporic candidates for each gene (PDIG_68680, PDIG_33760, PDIG_56860) was isolated and molecularly analyzed by Sanger sequencing (Fig. 2B-D) (Supp. Fig. S3). The percentage of editing efficiency was calculated for each of these three genes (Fig. 2E), and the type of mutations arising after DSBs and NHEJ-mediated repair was shown (Fig. 2F). In the case of PDIG_68680, around 54% of the colonies (13 out of 24) showed nucleotide insertions or deletions within the target gene sequence (Fig. 2B, E) generating in most of the cases (84.6%) frameshift mutations (Fig. 2F). For PDIG_33760, around 79% of the colonies showed gene editing within the target sequence (Fig. 2C, E) generating frameshift indels in approx. 60% of the strains, although in-frame indels (≈ 20%) and long indels (≈ 10%) were also found (Fig. 2F). A large insertion of > 50 bp was only identified in one mutant of PDIG_33760, showing a 62 bp insertion of a G-patch DNA repair protein gene (XM_014682453.1) and its upstream genomic sequence acting as a pseudo-donor template. Finally, for PDIG_56860, about 83% of the colonies showed nucleotide indels within the target gene (Fig. 2D, E) with frameshift indels accounting for 90% of the mutations observed (Fig. 2F). Once the edited mutants were identified, these strains were plated on non-selective plates to allow plasmid curation. After four streaks on non-selective plates, the strains lost their ability to grow in the presence of phleomycin. The absence of the phleomycin resistance cassette present in the AMA1 plasmids was additionally assessed by PCR of genomic DNA of the edited mutants (Supp. Fig. S2). Negative PCR results showed that the phleomycin resistance cassette was not integrated into the genome. However, we cannot discard the possibility that other (partial) integrations of the AMA1 vectors could have randomly occurred, which would have also resulted in the same negative PCR results. In this case, whole genome sequencing (WGS) would be the approach to search for the presence of partial fragments of the vectors genome-wide. Nevertheless, it is important to highlight that integration of AMA1-based plasmids is strange. No AMA1-based plasmid integration has been reported for several *Penicillium* species e.g., *Penicillium chrysogenum* [38], *P. subrubescens* [39] or *P. expansum* [21]. Moreover, in a recent study performed in *A. niger*, more than 90 CRISPR/Cas9-edited strains transformed with AMA1-derived plasmids were sequenced through WGS to find possible off-targets and plasmid integrations after strain curation [40]. In this study, no plasmid integrations were found regardless of their Non-Homologous End-Joining (NHEJ)- proficient or deficient genetic backgrounds, which would further support our results. Thus, since no plasmid integration was identified despite applying two additional rounds of selection in the presence of the antibiotic, this suggests that in the case of *P. digitatum* the probability of integration of pLM-AMA15.0 plasmid derivatives into the genome is very low, which greatly benefits their use in this organism.

## Conclusions

With this study, we demonstrate that additional rounds of transformant streaks under selection pressure conditions in a medium that promotes a slower growth rate of the fungus, as it is the case of PdMM, with increased incubation times improve CRISPR/Cas9 gene editing efficiency in *P. digitatum* from the previously reported 10% to up to 83% through the recyclable AMA1-based pLM-AMA15.0 plasmids. This relevant improvement of the editing efficiency together with the recyclable nature of the plasmid and selection used will allow for the efficient generation of *P. digitatum* single and -most importantly- multiple mutants aimed at studying, for instance, virulence gene functionality or antifungal resistance mechanisms without the limitation of selection markers. Moreover, the approach described in this study might help increase CRISPR/Cas9 gene editing efficiencies in other economically relevant *Penicillium* species for which the editing efficiency through similar CRISPR/Cas9 systems has been reported to be low, as it is the case of the enzyme producer *Penicillium subrubescens* [39] or the phytopathogen *P. expansum* [21], and likely in other relevant fungal species from genera other than *Penicillium*.

## Methods

Strains and growth conditions.

*P. digitatum* CECT 20796 (isolate PHI26) [3] was used as the parental strain. This strain and the transformants thereof generated through CRISPR/Cas9 technology were routinely cultured on PDA plates (Difco-BD Diagnostics) for 5–7 days at 25 °C. Conidia were harvested from the PDA plates with a fire-sterilized metal spatula, dispersed in sterile milli-Q H_2_O and concentration was determined and adjusted using a hemocytometer. Growth of monosporic transformants was analyzed depositing 5 µL of conidia suspension (5 × 10^4^ conidia/mL) in the center of PDA plates and colonies were analyzed by visual inspection. Vectors generated in this study were propagated in *Escherichia coli* JM109 grown in lysogeny broth (LB) medium [41] supplemented with 25 µg/mL chloramphenicol (Sigma-Aldrich) at 37 ºC.

Generation of DNA constructs.

Genes of interest (Table 1) were screened for CRISPR target sites using the Geneious Prime software version 2023.0.4 (https://www.geneious.com/), and the available *P. digitatum* CECT 20796 annotated genome [3, 16]. The sgRNA sites were specifically designed to target genes PDIG_53730 (*pksP*), PDIG_54100 (*arp2*), PDIG_68680 (*dfg5*), PDIG_33760 (*rodA*) and PDIG_56860, considering NGG as the protospacer adjacent motif (PAM) (Supp. Table S1). The 20 bp spacer sequences were selected considering no off-targets and high on-target activity predicted by the experimentally validated model described in [42].

Derivates of the self-replicative CRISPR/Cas9 plasmid pLM-AMA15.0 (AddGene ID #138,944) to target each gene were generated as described in [43]. The 20 bp spacer sequence defining each CRISPR target site was supplied as a separate DNA piece together with the hammerhead ribozyme (HH) sequence. DNA pieces were generated by PCR reaction (NZYTaq II, NZYTech^®^) with two overlapping primers and the resulting amplicons were purified (Wizard^®^ SV Gel and PCR Clean-Up System, Promega). The fragments were then cloned into the pLM-AMA15.0 plasmid through a ‘one-pot’ Golden Gate restriction-ligation reaction [44] with *Bsa*I restriction enzyme (*Bsa*I, ThermoFisher scientific) and T4 DNA ligase (Promega) with a vector/insert ratio of 1/100.

Protoplast generation, fungal transformation and mutant confirmation.

**Fig. 3:**
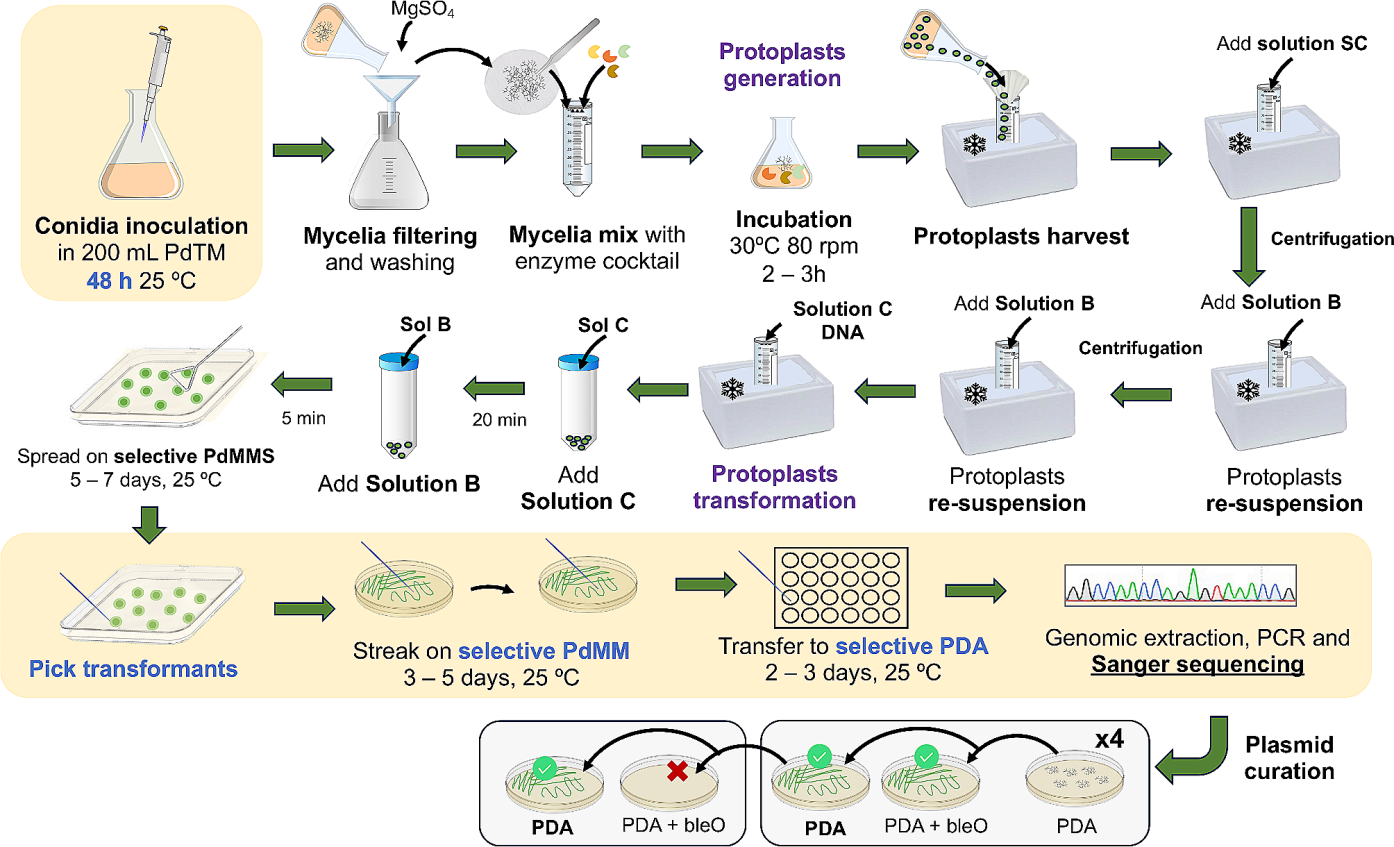
Schematic overview of the optimized *P. digitatum* protoplast transformation and mutant selection protocols. Modified steps from [23] are highlighted in yellow. Briefly, for protoplast generation, conidia were cultured in PdTM for 48 h instead of 24 h. After transformation, up to two additional selection steps were effectuated by streaking individual transformants on PdMM plates supplemented with the phleomycin antibiotic (bleO). See main text for more details

The transformation protocol was optimized based on the previously described method in [21] (Supp. Table S2). The optimized protocol for protoplast generation, transformation and transformant selection is summarized in Fig. 3 (main changes are highlighted in yellow boxes). For protoplasts generation, freshly harvested spores obtained from a 5-day old PDA plate at a concentration of 2 × 10^6^ conidia/mL were inoculated in 2 L plastic Erlenmeyer flasks containing 200 mL of *P. digitatum* transformation medium (PdTM) and maintained at 25 °C and 200 rpm for 48 h. Then, the culture was filtered through sterile Miracloth, washed with 0.6 M MgSO_4_ and dried by gentle squeezing between two sheets of UV-sterilized Miracloth paper. Resulting mycelia were resuspended in PS buffer with a ratio of 6.5 mL PS/g mycelium and mixed with the VinoTaste^®^ Pro lysing enzyme (Novozymes) (0.5 g enzyme/g mycelium in 15 mL PS). The mix was incubated in a rotary shaker at 30 °C and 80 rpm for 2–3 h, until rounded, non-extruded protoplasts were easily identified under the microscope. Protoplast suspensions were then placed on ice and filtered through sterile double-layer Miracloth paper. Cold SC solution was added to the protoplast suspensions to reach 45 mL of volume. Protoplasts were centrifuged 1700 ×*g* for 10 min at 4 °C and the pellet was washed with 10 mL solution B. The protoplast suspension was centrifuged again 750 ×*g* for 10 min at 4 ºC and it was finally re-suspended in solution B to reach a concentration of 1 × 10^7^ protoplasts/mL.

For transformation, 200 µL of protoplasts (1 × 10^7^ protoplasts/mL) were mixed with 50 µL of solution C and a maximum of 10 µL DNA solution containing 3 µg of each pLM-AMA15.0 plasmid. The mix was incubated on ice for 20 min. After incubation, 2 mL of solution C were added and, after 5 more min of incubation, 2 mL of solution B were poured to the protoplast suspension. Finally, protoplasts were spread over phleomycin (BleO)-containing square plates (35 µg/mL) of *P. digitatum* minimal medium sucrose (PdMMS) for regeneration and selection. For each experiment, two replicates were performed for each target gene. Additionally, untransformed protoplasts were spread over selective and non-selective plates as negative and regeneration controls of protoplast’s viability, respectively. All plates were incubated at 25 ºC until sporulated colonies were observed (between 5 and 7 days).

Transformants were picked and streaked in selective PdMM plates supplemented with phleomycin (35 µg/mL) for one (in the case of target genes with associated phenotypical changes) or two rounds (for genes without visual phenotypes). Single colonies were finally picked and transferred to 24-well PDA selective plates and genomic DNA was isolated for each colony (NZY Tissue gDNA Isolation kit, nzytech). Mutants were confirmed by PCR amplification (BIOTAQ™ DNA Polymerase, Bioline) and subsequent Sanger sequencing using primers indicated in Supp. Table S1. Multiple sequence alignments against parental gene sequences were effectuated with the Geneious Aligner (Geneious Prime^®^ 2023.0.4) to confirm gene editing. Finally, the verified CRISPR/Cas9 mutants were streaked on non-selective PDA plates for at least four rounds to cure the strains from the plasmid. Curation was confirmed by a last streak in selective PDA plates to verify their inability to grow in the presence of the phleomycin antibiotic and by PCR from genomic DNA of the cured mutants using specific oligonucleotides to amplify the phleomycin cassette present in the plasmid (Supp. Table S1).

### Electronic supplementary material

Below is the link to the electronic supplementary material.

Supplementary Material 1

Supplementary Material 2

Supplementary Material 3

## Data Availability

No datasets were generated or analysed during the current study.
