# Supplementary material for: Increasing the efficiency of CRISPR/Cas9-mediated genome editing in the citrus postharvest pathogen Penicillium digitatum
Source: Fungal Biol Biotechnol. 2024 Jul 13;11:8. doi: 10.1186/s40694-024-00179-0 (PMC11245846; doi:10.1186/s40694-024-00179-0)
Supplement: Supplementary file 1 — Supplementary Material 1 [file 40694_2024_179_MOESM1_ESM.pdf]

Supplementary Figures

Increasing the efficiency of CRISPR/Cas9-mediated genome editing in the citrus postharvest pathogen *Penicillium digitatum*

Carolina Ropero-Pérez, Jose F. Marcos, Paloma Manzanares and Sandra Garrigues*

Food Biotechnology Department, Instituto de Agroquímica y Tecnología de Alimentos (IATA), Consejo Superior de Investigaciones Científicas (CSIC), Catedrático Agustín Escardino Benlloch 7, 46980 Paterna, Valencia, Spain

* Correspondence: Sandra Garrigues: sgarrigues@iata.csic.es

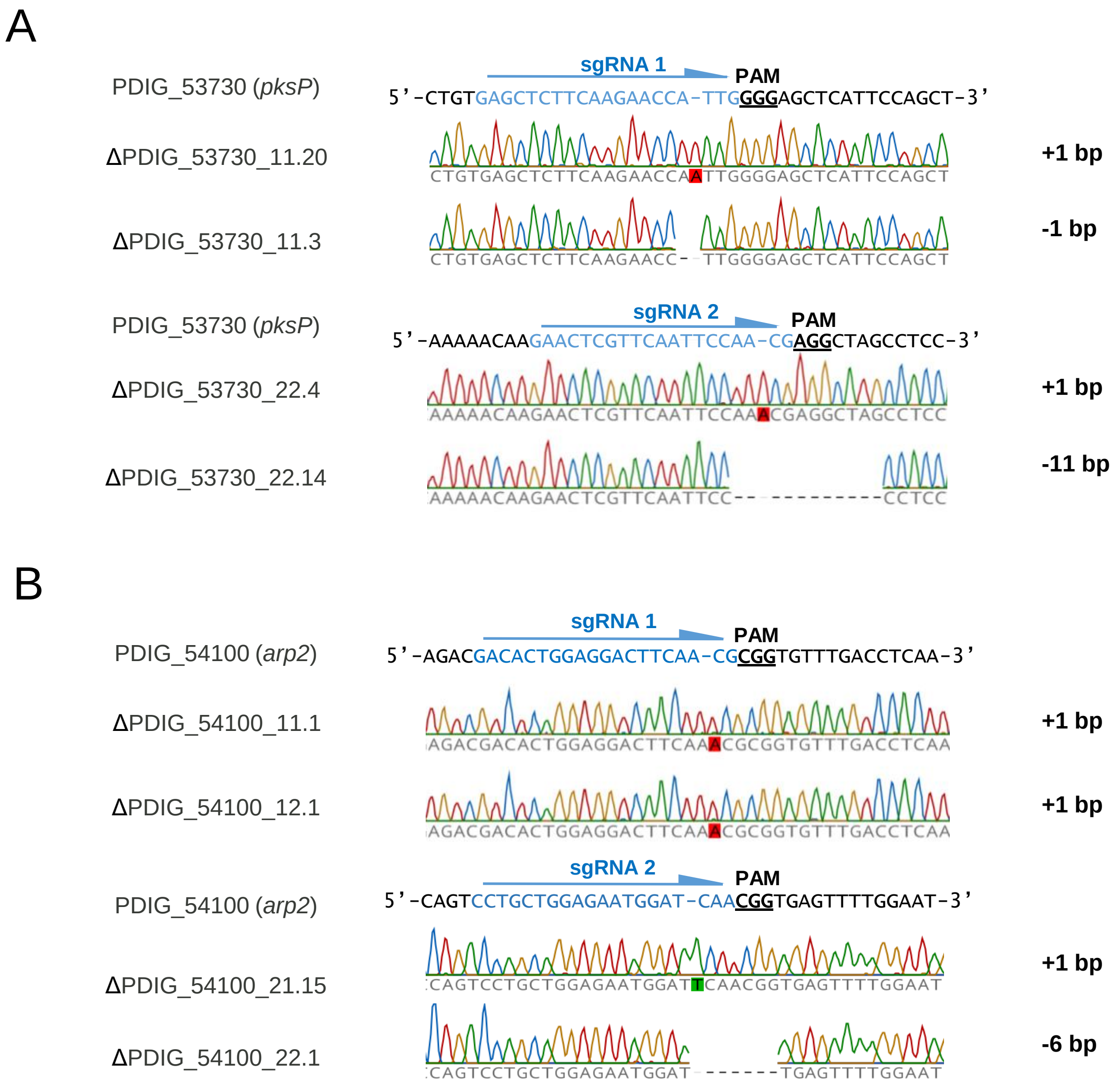

Supplementary Fig. S1: Nucleotide sequence alignments of Sanger sequencing results for PDIG_53730 (*pksP)* (A) and PDIG_54100 (*arp2*) (B) mutants compared to the parental sequences. Chromatograms and multiple sequence alignments were performed with Geneious Prime® 2023.0.4. PAM sequences are underlined and 20-bp protospacers are indicated in blue (sgRNA 1) and green (sgRNA 2).

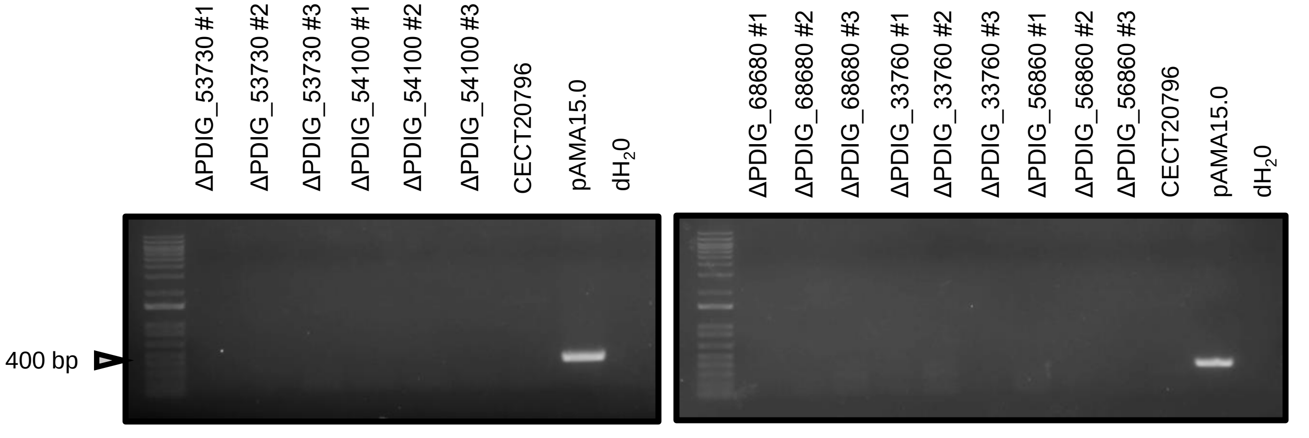

Supplementary Fig. S2: Molecular characterization of three independent *P. digitatum* cured mutants of the five genes under study (PDIG_53730, PDIG_54100, PDIG_68680, PDIG_33760, PDIG_56860) with primers Bleo_Fw and Bleo_Rv (Supp. Table S1). The 400 bp band corresponds to a fragment of the phleomycin resistence (*bleO*) casette present in the AMA15.0 plasmid. *P. digitatum* CECT20796 parental strain was included as negative control.

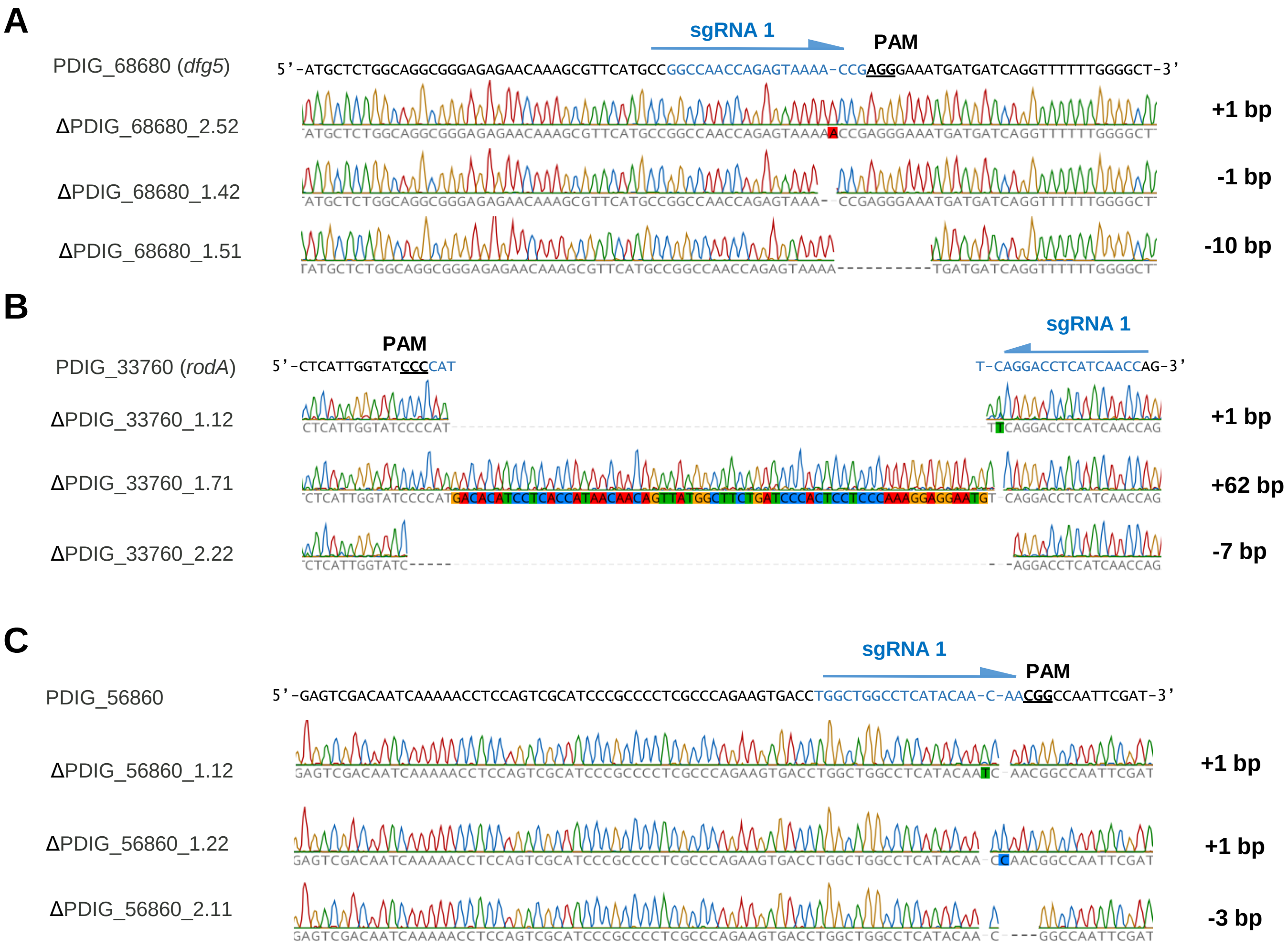

Supplementary Fig. S3: Nucleotide sequence alignments of Sanger sequencing results for PDIG_68680 (*dfg5*) (A), PDIG_33760 (*rodA*) (B) and PDIG_56860 (C) mutants compared to the parental sequences. Chromatograms and multiple sequence alignments were performed with Geneious Prime® 2023.0.4. PAM sequences are underlined and 20-bp protospacers for sgRNA 1 are indicated in blue.
