# Supplementary material for: Increasing the efficiency of CRISPR/Cas9-mediated genome editing in the citrus postharvest pathogen Penicillium digitatum
Source: Fungal Biol Biotechnol. 2024 Jul 13;11:8. doi: 10.1186/s40694-024-00179-0 (PMC11245846; doi:10.1186/s40694-024-00179-0)
Supplement: Supplementary file 2 — Supplementary Material 2 [file 40694_2024_179_MOESM2_ESM.docx]

**Supp. Table S1.** Primers used in this study.

| **Primer** | **Use^1^** | **Sequence 5’-3’^2^** | **Origin** | **Purpose** | **Target** | **Reference** |
| --- | --- | --- | --- | --- | --- | --- |
| OJM654 | F | CGACTCGGTGCCACTTTTTC | *E. coli* | screening | AMA15.0 backbone | [23] |
| OJM655 | R | CATCCATACTCCATCCTTCCC | *E. coli* | screening | AMA15.0 backbone | [23] |
| OJM758 | F | ATGGTCTCACCGAAGTGTCCTGATGAGTCCGTGAGGACGAAACGAG | *P. digitatum* | sgRNA1 | PDIG_54100 | This study |
| OJM759 | R | ATGGTCTCTAAACCGTTGAAGTCCTCCAGTGTCGACGAGCTTACTCGTTTCGTCCTCACGGACTCA | *P. digitatum* | sgRNA1 | PDIG_54100 | This study |
| OJM760 | F | ATGGTCTCACCGAAGCAGGCTGATGAGTCCGTGAGGACGAAACGAG | *P. digitatum* | sgRNA2 | PDIG_54100 | This study |
| OJM761 | R | ATGGTCTCTAAACTTGATCCATTCTCCAGCAGGGACGAGCTTACTCGTTTCGTCCTCACGGACTCA | *P. digitatum* | sgRNA2 | PDIG_54100 | This study |
| OJM778 | F | CACTCCTTGACAACCTTTCAC | *P. digitatum* | Sequencing | PDIG_54100 | This study |
| OJM779 | R | GTCAATTAGATGTTAGCTCCAC | *P. digitatum* | Sequencing | PDIG_54100 | This study |
| OJM780 | R | GAGCAATCGCATTCACAGTAG | *P. digitatum* | Sequencing | PDIG_54100 | This study |
| OJM781 | F | GAACGCTTACGACTACATCCAG | *P. digitatum* | Sequencing | PDIG_54100 | This study |
| OJM782 | F | ATGGTCTCACCGAGAGCTCCTGATGAGTCCGTGAGGACGAAACGAG | *P. digitatum* | sgRNA1 | PDIG_53730 | This study |
| OJM783 | R | ATGGTCTCTAAACCAATGGTTCTTGAAGAGCTCGACGAGCTTACTCGTTTCGTCCTCACGGACTCA | *P. digitatum* | sgRNA1 | PDIG_53730 | This study |
| OJM784 | F | ATGGTCTCACCGAGAGTTCCTGATGAGTCCGTGAGGACGAAACGAG | *P. digitatum* | sgRNA2 | PDIG_53730 | This study |
| OJM785 | R | ATGGTCTCTAAACCGTTGGAATTGAACGAGTTCGACGAGCTTACTCGTTTCGTCCTCACGGACTCA | *P. digitatum* | sgRNA2 | PDIG_53730 | This study |
| OJM790 | F | GTCAGGTGTATCTGTTCGGAG | *P. digitatum* | Sequencing | PDIG_53730 | This study |
| OJM791 | R | GGACCACTTATCGTGAGTCC | *P. digitatum* | Sequencing | PDIG_53730 | This study |
| OJM792 | F | GATCAACCACAACTTTCCAACAG | *P. digitatum* | Sequencing | PDIG_53730 | This study |
| OJM793 | R | GTAACCACAGGGCTATGCTCATC | *P. digitatum* | Sequencing | PDIG_53730 | This study |
| OJM814 | F | ATGGTCTCACCGATTGGCCCTGATGAGTCCGTGAGGACGAAACGAG | *P. digitatum* | sgRNA | PDIG_68680 | This study |
| OJM815 | R | ATGGTCTCTAAACCGGTTTTACTCTGGTTGGCCGACGAGCTTACTCGTTTCGTCCTCACGGACTCA | *P. digitatum* | sgRNA | PDIG_68680 | This study |
| OJM818 | F | ATGGTCTCACCGATCAACCCTGATGAGTCCGTGAGGACGAAACGAG | *P. digitatum* | sgRNA | PDIG_33760 | This study |
| OJM819 | R | ATGGTCTCTAAACCATTCAGGACCTCATCAACCGACGAGCTTACTCGTTTCGTCCTCACGGACTCA | *P. digitatum* | sgRNA | PDIG_33760 | This study |
| OJM820 | F | ATGGTCTCACCGACAGCCACTGATGAGTCCGTGAGGACGAAACGAG | *P. digitatum* | sgRNA | PDIG_56860 | This study |
| OJM821 | R | ATGGTCTCTAAACTTGTTGTATGAGGCCAGCCAGACGAGCTTACTCGTTTCGTCCTCACGGACTCA | *P. digitatum* | sgRNA | PDIG_56860 | This study |
| OJM828 | F | CTGTGCAAGCGATAGAGCTG | *P. digitatum* | Sequencing | PDIG_68680 | This study |
| OJM829 | R | CTTGCACTCAGTGAGGTCAC | *P. digitatum* | Sequencing | PDIG_68680 | This study |
| OJM832 | F | GTCTGGACAACTCACAGACCG | *P. digitatum* | Sequencing | PDIG_33760 | This study |
| OJM833 | R | TCTCCGAGTGCTACCATACG | *P. digitatum* | Sequencing | PDIG_33760 | This study |
| OJM834 | F | ATGGTCTTGCCGTAACTGG | *P. digitatum* | Sequencing | PDIG_56860 | This study |
| OJM835 | R | CCTCGTTATCGTACTTCGAGC | *P. digitatum* | Sequencing | PDIG_56860 | This study |
| Bleo_Fw | F | AAGTTGACCAGTGCCGTTCC | *S. hindustanus* | screening | *bleO* from AMA15.0 backbone | This study |
| Bleo_Rv | R | TCAGTCCTGCTCCTCGG | *S. hindustanus* | screening | *bleO* from AMA15.0 backbone | This study |

^1^ F, forward; R, reverse.

^2^ the 20 bp for sgRNA design are underlined
