# Supplementary material for: Increasing the efficiency of CRISPR/Cas9-mediated genome editing in the citrus postharvest pathogen Penicillium digitatum
Source: Fungal Biol Biotechnol. 2024 Jul 13;11:8. doi: 10.1186/s40694-024-00179-0 (PMC11245846; doi:10.1186/s40694-024-00179-0)
Supplement: Supplementary file 3 — Supplementary Material 3 [file 40694_2024_179_MOESM3_ESM.docx]

**Supplementary Table S2**. Media and solutions used for protoplasts generation and transformation. Adapted from [23]

| **Description** | **Composition a** |
| --- | --- |
| **Sorbitol Calcium Chloride (SC) solution** | 18.2% sorbitol, 0.74% CaCl2 * 2H2O |
| **Phosphate Sorbitol (PS) buffer** | 0.2 M sodium phosphate buffer, 0.8 M sorbitol, pH 6 |
| **Solution B** | 1 M sorbitol, 50 mM CaCl2, 10 mM Tris−HCl, pH 7 |
| **Solution C** | 25% PEG 6000, 50 mM CaCl2, 10 mM Tris−HCl, pH 7.5 |
| ***Penicillium digitatum* Transformation Medium (PdTM**) | 0.6% of NaNO3, 0.15% KH2PO4, 0.05% KCl, 0.05% MgSO4, 200 μL/L of ***Aspergillus* trace elements** (1% EDTA, 0.44% ZnSO4 · 7H2O, 0.1% MnCl2 · 4H2O, 0.032% CoCl2 · 6H2O, 0.032% CuSO4 · 5H2O, 0.022% (NH4)6Mo7O24 · 4H2O, 0.15% CaCl2 · 2H2O, 0.1% FeSO4 · 7H2O), 0.5% yeast extract, 0.2% casamino acids, 2% D-glucose, 0.08% NH4NO3. |
| ***Penicillium digitatum* minimal medium sucrose (PdMMS)** | 1% D-glucose, 0.0001% FeSO4, 0.08% NH4NO3, 2% **salt solution B** (1.5% NaCl, 2.6% MgSO4 x 7 H2O, 7.6% KH2PO4), 1% **trace elements solution B** (0.01% H3BO3, 0.01% CuSO4 x 5H2O, 0.01% MnSO4 x H2O, 0.01% Na2MoO4 x 2H2O, 0.01% ZnSO4 x 7 H2O), 40 mM MES buffer (pH 5.3), 0.95 M sucrose,  1.5% agar. |

a Percent values are given as w/v for solids and v/v for solutions.
